# Association between the alkaline phosphatase-to-albumin ratio and recorded early all-cause mortality within 90 days of admission in patients with heart failure: a retrospective cohort study in a Chinese population

**DOI:** 10.3389/fcvm.2026.1936612

**Published:** 2026-09-16

**Authors:** Zhenyu Yuan, Mohan Li, Lei Zhao, Hong Geng, Qiang Ji, Kun Zuo, Yuanzhi Luo, Guanli Liang, Gengxu He

**Affiliations:** 1 Department of Thoracic and Cardiovascular Surgery, The First Affiliated Hospital of Hebei North University, Zhangjiakou, China; 2 Department of Pediatrics/Neonatology, Hebei General Hospital, Shijiazhuang, China

**Keywords:** 90-day mortality, alkaline phosphatase-to-albumin ratio, early mortality, heart failure, risk marker

## Abstract

**Background:**

APAR combines alkaline phosphatase and albumin, two routine laboratory measurements. We examined its association with recorded early all-cause mortality within 90 days of admission in hospitalized patients with heart failure (HF), prioritizing continuous APAR over a mortality-derived cutoff.

**Methods:**

We analyzed version 1.3 of the Zigong Heart Failure Database. Among 2,008 patients, 1,906 had calculable APAR and a recorded vital-status outcome within 90 days; 37 deaths were recorded. Exact event times were available for 1,905 patients (36 deaths). The primary model assessed standardized ln(APAR), adjusting for the eight released age categories as a 1–8 ordinal score, sex, log(BNP), eGFR, sodium, and CCI, with NYHA class stratification. Mortality timing, post-discharge survival, hemoglobin adjustment, and E-values were examined in sensitivity analyses.

**Results:**

Recorded all-cause mortality within 90 days of admission was 1.94%. In the complete-case model (*n* = 1,824; 36 deaths), each 1-SD increase in ln(APAR) was associated with higher mortality (HR 1.375, 95% CI 1.024–1.846; *P* = 0.034), whereas raw APAR per 1 U/g was not (HR 1.146, 95% CI 0.988–1.330; *P* = 0.072). The spline showed an overall association (*P* = 0.025) and nonlinearity (*P* = 0.023). Nine of 37 deaths were confirmed in-hospital. Among 1,897 patients discharged without recorded in-hospital death, 28 died within 90 days: 15 on a later calendar date, 12 on the discharge date, and one with missing exact timing. In the strict post-discharge analysis, the adjusted HR was 1.460 (95% CI 0.931–2.289; *P* = 0.099; 15 events). APAR showed modest discrimination (AUC 0.698), while incremental value was limited: *Δ*C-index 0.018 (*P* = 0.104), IDI 0.0019 (95% CI −0.0035 to 0.0252), small decision-curve gains, and continuous NRI 0.179 (95% CI −0.129 to 0.577).

**Conclusion:**

Higher admission ln(APAR) was associated with recorded early all-cause mortality within 90 days of admission, but the association was modest, nonlinear, scale-dependent, and based on few events. Post-discharge estimates were directionally consistent but imprecise. External validation is required before APAR or any cutoff is used for clinical risk stratification.

## Introduction

Heart failure (HF) remains a major cause of death, recurrent hospitalization, and healthcare use worldwide (1–5). The period after hospitalization is particularly vulnerable, with a substantial concentration of adverse events soon after discharge (6–8). Age, hemodynamic severity, renal dysfunction, congestion, nutritional reserve, and comorbidity all contribute to early risk (9). Although established models such as MAGGIC and the Seattle Heart Failure Model combine several of these domains (10, 11), simple laboratory markers may still be useful when they add information without requiring additional testing.

The heart and liver are closely linked in HF. Venous congestion and reduced perfusion can alter liver tests, while systemic inflammation and hemodilution may affect both hepatic markers and serum proteins (12, 13). Albumin is influenced by hepatic synthesis, nutritional status, inflammation, fluid balance, and systemic disease, and lower concentrations have been associated with poorer HF outcomes (14–16). Nutritional depletion and wasting are themselves associated with adverse prognosis in HF (17, 18). Alkaline phosphatase (ALP), in turn, reflects hepatobiliary and bone metabolism and has been linked to vascular calcification, mineral metabolism, inflammation, and cardiovascular mortality (19–26).

APAR is simply ALP divided by albumin. Higher APAR has been associated with adverse outcomes in coronary artery disease, sepsis, and chronic kidney disease (27–29), but those observations do not show that the ratio is superior to either component or that a data-derived cutoff can be transferred between populations. Evidence in hospitalized HF is limited. We therefore examined the association between admission APAR and recorded early all-cause mortality within 90 days of admission. We focused first on APAR as a continuous exposure, then examined its nonlinear pattern, compared it formally with ALP and albumin, and tested whether it added measurable information to a small clinical model. We also used sensitivity analyses to address missing data, limited event counts, and non-HF causes of abnormal ALP or albumin.

## Materials and methods

### Study population and data source

We used the Zigong Heart Failure Database, a restricted-access PhysioNet resource derived from consecutive patients hospitalized with HF at Zigong Fourth People's Hospital, Sichuan Province, China, between December 2016 and June 2019 (30, 31). We used database version 1.3. The source dataset includes demographic information, admission clinical findings, laboratory measurements, comorbidities, medications, and externally obtained follow-up outcomes. Follow-up in the source study was scheduled at 28 days, 3 months, and 6 months, with telephone follow-up when an in-person visit was not possible (30, 31).

The source cohort contained 2,008 patients. We excluded 102 because ALP or albumin was unavailable, leaving 1,906 patients with calculable APAR and a nonmissing 90-day vital-status field. One patient was recorded as having died within 90 days but had no exact event date. We retained that patient in fixed-horizon analyses but excluded the patient from Cox, Kaplan–Meier, and restricted cubic spline analyses, leaving 1,905 patients and 36 exact-time deaths for survival analyses. The primary multivariable Cox model included 1,824 complete cases; all 36 exact-time deaths remained in that model (Figure 1).

**Figure 1:**
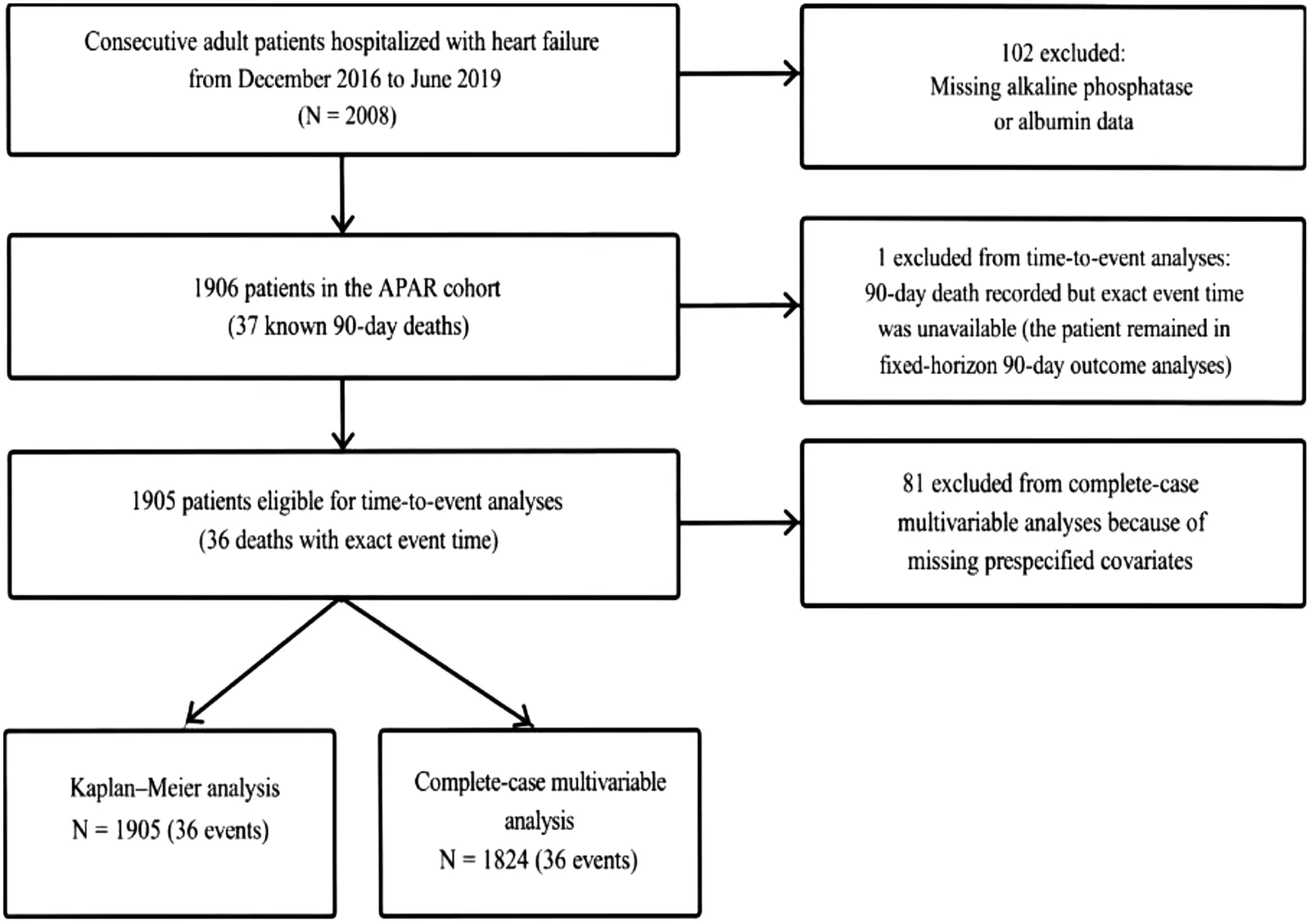
Flowchart of patient selection and analytical cohorts.

This secondary analysis used de-identified data. No prospective protocol for the present analysis was registered. We specified the primary exposure and a clinically motivated, event-constrained adjustment set before fitting the reported models; we did not use automated stepwise selection or univariable *P*-value screening. We reported the study with reference to STROBE, relevant TRIPOD items, and REMARK principles for prognostic-marker reporting (32–34).

### Exposure definition

We calculated APAR as ALP (U/L) divided by serum albumin (g/L), giving units of U/g. We used the admission values released in the database. Because APAR was right-skewed and a same-cohort outcome-derived cutoff can overstate an association, our primary exposure was ln(APAR), standardized so that the main HR represents a 1-SD increase. We also examined raw APAR per unit and per SD, and used APAR tertiles as a secondary categorical analysis with a trend test.

As an exploratory analysis, we also estimated a Youden cutoff from the 90-day outcome. The apparent cutoff was 2.82 U/g, and we assessed its sampling variability and optimism with 1,000 bootstrap resamples. The public data documentation does not specify the ALP analytical platform, reagent manufacturer, albumin assay method, or ALP isoenzyme measurements. We therefore regard this cutoff as laboratory- and cohort-specific rather than a transferable clinical boundary.

### Outcome ascertainment

The primary outcome was recorded all-cause mortality within 90 days of the index admission. Time zero for the primary survival analysis was the admission date, and deaths during the index hospitalization were included if they occurred within 90 days. The released 90-day status field was nonmissing in all 1,906 patients with calculable APAR. A confirmed in-hospital death was defined strictly by the released hospital_outcome field as Dead; discharge_destination was used only for quality-control review because a small number of records showed disagreement between disposition fields. Among patients discharged without a recorded in-hospital death, exact-time deaths were classified as later-day post-discharge when death_time_days exceeded discharge_day, as same-day when the two were equal, and as a temporal conflict when death_time_days was earlier than discharge_day. Same-day events were left unassigned to either side of discharge because the public release provides calendar-day rather than within-day timing.

For a strict post-discharge sensitivity analysis, time zero was reset to discharge. We excluded same-day deaths with unresolved within-day ordering, deaths without an exact event time, records with discharge-status quality-control conflicts, and records without positive follow-up time before day 90. Surviving patients were censored at 90 days after the index admission. The public release does not provide patient-level logs of unsuccessful contact attempts or a separate category for unresolved follow-up, so we continued to rely on the released follow-up outcome fields rather than inferring survival from discharge disposition (Supplementary Table S8).

### Covariates and missing data

Given the 36 deaths with exact event times, the primary model was kept parsimonious. The released age variable comprised eight ordered categories: (21,29], (29,39], (39,49], (49,59], (59,69], (69,79], (79,89], and (89,110] years. Age was represented by a single ordinal score from 1 to 8, preserving the full ordering while limiting model degrees of freedom. A seven-degree-of-freedom factor specification produced separation and unstable coefficients because two age categories had no deaths and several contained only one event. The remaining primary covariates were sex, log-transformed BNP, eGFR, serum sodium, and CCI, with NYHA class as a stratification factor (Supplementary Table S9).

These covariates represent demographic, HF-severity, renal, electrolyte, and comorbidity domains established in HF prognosis (9–11, 35–37). We used eGFR rather than BUN in the primary renal adjustment. ALP and albumin were not entered as ordinary covariates in the primary APAR model because they are mathematical components of the exposure. An additional sensitivity model included hemoglobin to assess whether the APAR association was sensitive to anemia-related confounding.

We summarized missingness for all reported variables and compared patients with and without calculable APAR (Supplementary Tables S1–S2). The primary Cox analysis was complete-case. As a sensitivity analysis, we used multiple imputation with 20 datasets for missing primary-model covariates. Additional analyses adjusted for HF treatment burden, diuretic exposure, liver disease and malignancy, liver biochemistry, hemoglobin, and LVEF where available; we also repeated analyses after excluding liver disease and/or malignancy. Weight was summarized from the released values as median (IQR). BMI was not included in inferential analyses because the released height/weight fields yielded implausible extreme BMI values; the anthropometric distribution is summarized in Supplementary Table S11.

### Statistical analysis

We summarized continuous variables as mean ± SD or median (IQR), as appropriate, and categorical variables as *n* (%). Weight was summarized as median (IQR). Baseline comparisons across APAR tertiles were accompanied by the largest pairwise standardized mean difference (SMD); *P* values in Table 1 are descriptive. Kaplan–Meier curves were compared with the log-rank test (38). Cox regression provided HRs and 95% CIs (39). Proportional-hazards assumptions were assessed with scaled Schoenfeld residuals and global testing; NYHA class was stratified in the primary model.

**Table 1:** Baseline characteristics of patients with heart failure according to APAR tertiles.

Variable	T1 (*n* = 636)	T2 (*n* = 635)	T3 (*n* = 635)	*P* value	Max |SMD|	Missing *n*
Age category				0.039	0.189	0
(21, 29]	1 (0.2)	1 (0.2)	2 (0.3)			
(29, 39]	3 (0.5)	5 (0.8)	4 (0.6)			
(39, 49]	23 (3.6)	16 (2.5)	11 (1.7)			
(49, 59]	30 (4.7)	33 (5.2)	39 (6.1)			
(59, 69]	126 (19.8)	111 (17.5)	110 (17.3)			
(69, 79]	251 (39.5)	213 (33.5)	217 (34.2)			
(79, 89]	183 (28.8)	224 (35.3)	210 (33.1)			
(89, 110]	19 (3.0)	32 (5.0)	42 (6.6)			
Sex				0.877	0.019	0
Female	370 (58.2%)	374 (58.9%)	365 (57.5%)			
Male	266 (41.8%)	261 (41.1%)	270 (42.5%)			
Weight	51.00 (45.00, 60.00)	50.00 (45.00, 60.00)	50.00 (45.00, 60.00)	0.027	0.121	0
Heart rate	83.83 ± 19.32	85.28 ± 21.27	86.71 ± 23.57	0.057	0.090	0
SBP	131.19 ± 23.77	132.54 ± 25.00	130.46 ± 25.09	0.311	0.056	0
DBP	76.73 ± 13.40	77.51 ± 14.86	75.77 ± 14.85	0.099	0.080	0
NYHA class				0.017	0.127	0
II	120 (18.9)	111 (17.5)	104 (16.4)			
III	346 (54.4)	337 (53.1)	306 (48.2)			
IV	170 (26.7)	187 (29.4)	225 (35.4)			
GCS	15.00 (15.00, 15.00)	15.00 (15.00, 15.00)	15.00 (15.00, 15.00)	0.001	0.100	0
CCI	2.00 (1.00, 2.00)	2.00 (1.00, 3.00)	2.00 (1.00, 3.00)	0.020	0.086	5
Diabetes				0.046	0.093	0
No	486 (76.4%)	508 (80.0%)	471 (74.2%)			
Yes	150 (23.6%)	127 (20.0%)	164 (25.8%)			
Chronic pulmonary disease				0.619	0.035	0
No	555 (87.3%)	562 (88.5%)	565 (89.0%)			
Yes	81 (12.7%)	73 (11.5%)	70 (11.0%)			
Chronic kidney disease				0.201	0.093	2
No	504 (79.2)	482 (75.9)	469 (73.9)			
Yes	132 (20.8)	152 (23.9)	165 (26.0)			
Liver disease				0.020	0.122	1
No	611 (96.1)	618 (97.3)	595 (93.7)			
Yes	25 (3.9)	17 (2.7)	39 (6.1)			
Any malignancy				<0.001	0.121	0
No	629 (98.9%)	629 (99.1%)	612 (96.4%)			
Yes	7 (1.1%)	6 (0.9%)	23 (3.6%)			
WBC	6.36 (4.98, 8.29)	6.52 (5.06, 8.56)	6.71 (5.14, 9.30)	0.025	0.152	21
Neutrophils	4.68 (3.45, 6.32)	4.84 (3.65, 6.60)	5.08 (3.71, 7.33)	0.001	0.176	21
Hemoglobin	118.67 ± 22.27	114.02 ± 24.76	111.86 ± 25.20	<0.001	0.190	22
Platelets	129.00 (97.00, 168.00)	137.50 (105.00, 177.00)	138.00 (98.75, 181.00)	0.018	0.124	21
ALP	59.00 (52.00, 66.00)	81.00 (73.00, 89.00)	116.00 (99.50, 139.00)	<0.001	1.622	0
Albumin	38.68 ± 4.15	36.79 ± 4.32	34.13 ± 5.29	<0.001	0.651	0
APAR	1.55 (1.37, 1.72)	2.20 (2.06, 2.37)	3.28 (2.89, 4.15)	<0.001	3.155	0
BUN	7.51 (5.76, 10.16)	7.75 (5.72, 11.39)	9.12 (6.44, 13.39)	<0.001	0.242	13
Creatinine	84.10 (65.33, 110.85)	87.30 (64.50, 123.00)	93.15 (65.97, 138.25)	0.001	0.192	13
eGFR	68.48 (47.66, 91.55)	65.38 (40.52, 89.45)	57.40 (36.50, 88.38)	<0.001	0.101	51
Potassium	3.92 ± 0.62	3.98 ± 0.70	4.04 ± 0.79	0.006	0.121	7
Sodium	138.89 ± 4.31	138.40 ± 4.86	137.63 ± 5.39	<0.001	0.172	7
Chloride	102.22 ± 5.50	102.01 ± 5.65	101.45 ± 6.76	0.065	0.084	7
BNP	570.67 (217.38, 1,306.49)	747.06 (295.28, 1,605.27)	1,123.28 (468.48, 2,358.95)	<0.001	0.304	25
Beta-blocker use				0.071	0.105	1
No	374 (58.8)	401 (63.1)	418 (65.8)			
Yes	261 (41.0)	234 (36.9)	217 (34.2)			
Diuretic use				0.248	0.076	1
No	15 (2.4)	9 (1.4)	7 (1.1)			
Yes	620 (97.5)	626 (98.6)	628 (98.9)			
Nitrate use				0.466	0.067	1
No	489 (76.9)	473 (74.5)	490 (77.2)			
Yes	146 (23.0)	162 (25.5)	145 (22.8)			
Vasoactive-drug use				<0.001	0.175	1
No	394 (61.9)	374 (58.9)	316 (49.8)			
Yes	241 (37.9)	261 (41.1)	319 (50.2)			
Digoxin use				0.487	0.064	1
No	311 (48.9)	318 (50.1)	332 (52.3)			
Yes	324 (50.9)	317 (49.9)	303 (47.7)			
90-day mortality				0.001	0.132	0
No	631 (99.2%)	626 (98.6%)	612 (96.4%)			
Yes	5 (0.8%)	9 (1.4%)	23 (3.6%)			

Values are mean ± SD, median (IQR), or *n* (%), as appropriate. *P* values are descriptive. Max |SMD| is the largest pairwise standardized mean difference across tertiles; Missing *n* is the number missing among all 1,906 patients. Weight is shown as median (IQR). BMI was not used for inference because implausible extreme anthropometric values were present in the public release (Supplementary Table S11). Units: weight, kg; heart rate, beats/min; SBP/DBP, mmHg; WBC/neutrophils/platelets, ×10^9^/L; hemoglobin/albumin, g/L; ALP/AST/ALT/GGT, U/L; APAR, U/g; BUN, mmol/L; creatinine/total bilirubin, μmol/L; eGFR, mL/min/1.73 m^2^; electrolytes, mmol/L; BNP, pg/mL.

We modeled nonlinearity with a restricted cubic spline using three knots at the 10th, 50th, and 90th percentiles of APAR (1.408, 2.198, and 3.897 U/g), with 2.198 U/g as the reference. We tested the overall APAR contribution by a likelihood-ratio comparison of the spline model with the covariate-only model (2 df) and tested nonlinearity by comparing the spline model with the corresponding linear APAR model (1 df). We displayed the exposure distribution with a rug. For discrimination, we calculated 90-day ROC AUCs and used DeLong tests for APAR vs. ALP and albumin (40). A cumulative/dynamic 90-day AUC was also calculated in the exact-time cohort; because the prepared survival data contained no censoring before day 90 among non-events, it was essentially the same as the fixed-horizon estimate.

To examine whether the ratio contributed information beyond its components, we fitted adjusted models for ln(APAR), ln(ALP), and ln(albumin) and compared an unrestricted ln(ALP) + ln(albumin) model with the constrained ratio form by likelihood-ratio test. We then compared the clinical model with the same model plus standardized ln(APAR) using Harrell C-index, 90-day AUC, likelihood-ratio testing, IDI, calibration, Brier score, decision-curve analysis, and continuous NRI (41, 42). Because reclassification metrics can appear favorable despite little change in absolute risk or clinical utility, IDI, calibration, Brier score, and decision curves were interpreted together, and continuous NRI was treated as a secondary metric rather than the lead evidence for incremental value. Bootstrap resampling was used for internal uncertainty assessment.

We used ridge-penalized Cox regression as a shrinkage sensitivity analysis. We also calculated an E-value for the adjusted ln(APAR) estimate and for the confidence-limit value closest to the null, using the HR as an approximate risk ratio; this analysis quantifies robustness to unmeasured confounding but does not directly model outcome misclassification (Supplementary Table S10). The strict post-discharge Cox model used the same clinical adjustment set, but its fully adjusted estimate was considered exploratory because only 15 definite later-day deaths remained. Exploratory subgroup analyses used unadjusted Cox models on the same 1-SD ln(APAR) scale and reported subgroup-specific HRs, interaction *P* values, and Benjamini-Hochberg FDR-adjusted q values. We repeated the primary model over 28 days because deaths were concentrated early. All tests were two-sided. We used R 4.5.1 with survival 3.8–3, rms 8.0–0, Hmisc 5.2-3, pROC 1.19.0.1, mice 3.18.0, glmnet 4.1–10, and riskRegression 2026.02.13.

## Results

### Cohort and baseline characteristics

Of 2,008 eligible patients, 1,906 had calculable APAR and recorded 90-day status. Thirty-seven patients had recorded all-cause mortality within 90 days of admission (1.94%). One death had no exact event date, leaving 1,905 patients and 36 deaths for time-to-event analyses; the primary complete-case model included 1,824 patients and retained all 36 deaths (Figure 1). Among the 36 exact-time deaths, the median time to death was 4.5 days (IQR, 2.0–17.2). Twenty-one occurred on days 1–7, four on days 8–14, seven on days 15–28, two on days 29–36, one on days 37–60, and one on days 61–90 (Supplementary Figure S1).

Nine patients were confirmed to have died during the index hospitalization (9/1,906, 0.47%). The remaining 1,897 patients had no recorded in-hospital death; 28 of them had a recorded 90-day death (1.48%). Of these 28 deaths, 15 occurred on a later calendar date after discharge, 12 were recorded on the same calendar day as discharge and therefore could not be ordered within that day, and one lacked an exact death time. No death was recorded on a calendar day earlier than discharge. Median index length of stay was 8.0 days (IQR, 6.0–10.0) overall and 17.0 days (IQR, 5.0–26.0) among confirmed in-hospital deaths. For context, the source database descriptor reported 14 in-hospital deaths among 2,008 patients (0.70%), which is a different outcome window from 90-day mortality (Supplementary Table S8).

The APAR tertiles contained 636, 635, and 635 patients (Table 1). Higher tertiles were characterized by lower albumin, hemoglobin, eGFR, and sodium and by higher ALP, GGT, BUN, creatinine, and BNP; NYHA IV and vasoactive-drug use were also more common in the highest tertile. The eight age categories are displayed directly in Table 1. Median weight was 51 (45–60), 50 (45–60), and 50 (45–60) kg across T1–T3, respectively; the first and third quartiles were identical across tertiles, consistent with heaping in the released weight values. We interpreted the overall baseline pattern as evidence that APAR tracks a broader severity profile rather than proof of a specific nutritional or inflammatory mechanism. Missingness was low for the primary-model variables, whereas LVEF and AST were substantially missing (Supplementary Table S1). Patients excluded because APAR could not be calculated differed in several baseline variables, including eGFR, sodium, and BNP, so some selection related to exposure availability cannot be excluded (Supplementary Table S2).

### Primary association and dose-response pattern

In the unadjusted model, a 1-SD increase in ln(APAR) was associated with 90-day mortality (HR, 1.637, 95% CI, 1.293–2.072; *P* < 0.001). After adjustment for the eight-category ordinal age score, sex, log(BNP), eGFR, sodium, and CCI, with NYHA class stratification, the HR was 1.375 (95% CI, 1.024–1.846; *P* = 0.034; *n* = 1,824; 36 deaths) (Table 2). On the raw scale, the adjusted HR was 1.146 per 1 U/g increase (95% CI: 0.988–1.330; *P* = 0.072). The difference in statistical significance across scales reinforces that the association should not be presented as a scale-invariant effect. We found no evidence of violation of the proportional-hazards assumption for ln(APAR) (*P* = 0.348), the ordinal age term (*P* = 0.320), or globally (*P* = 0.325; Supplementary Table S3).

**Table 2:** Association between APAR and 90-day all-cause mortality in Cox proportional hazards models.

Variable	Model 1 (Crude)		Model 2 (Adjusted)	
	HR (95% CI)	*P* value	HR (95% CI)	*P* value
Primary continuous analysis				
ln(APAR) per 1 SD	1.637 (1.293–2.072)	<0.001	1.375 (1.024–1.846)	0.034
Alternative continuous scales				
APAR per 1 U/g	1.093 (1.024–1.167)	0.008	1.146 (0.988–1.330)	0.072
APAR per 1 SD	1.142 (1.032–1.264)	0.008	1.233 (0.983–1.547)	0.072
ln(APAR) per 1 unit	3.23 (1.84–5.70)	<0.001	2.154 (1.059–4.382)	0.034
APAR tertiles				
T1	1.00 (Ref)		1.00 (Ref)	
T2	1.80 (0.60–5.37)	0.292	1.51 (0.50–4.52)	0.461
T3	4.50 (1.70–11.89)	0.002	2.67 (0.98–7.27)	0.055
Per-tertile trend		<0.001		0.036

Model 1 is unadjusted (*N* = 1,905; 36 deaths). Model 2 is adjusted for the eight released age categories represented by an ordinal score from 1 to 8, sex, standardized log(BNP), standardized eGFR, standardized serum sodium, and CCI, with NYHA functional class used as a stratification factor (*N* = 1,824; 36 deaths). The primary exposure is ln(APAR) per 1-SD increase; raw APAR results show the scale dependence of the association, and tertile/cutoff analyses are secondary or exploratory.

In the tertile analysis, the adjusted HRs were 1.51 for T2 vs. T1 (95% CI: 0.50–4.52; *P* = 0.461) and 2.67 for T3 vs. T1 (95% CI: 0.98–7.27; *P* = 0.055), with *P* for trend = 0.036. Kaplan–Meier curves showed the clearest separation for T3 (log-rank *P* = 0.001; Figure 2). The spline showed an overall APAR association (LR *χ*^2^ = 7.378, df = 2; *P* = 0.025) and evidence of nonlinearity (LR *χ*^2^ = 5.153, df = 1; *P* = 0.023). Relative to APAR 2.198 U/g, estimated risk rose mainly through the lower-to-middle range and then changed little at higher values; confidence intervals widened in the upper tail as observations became sparse (Figure 3). At the lowest APAR values, the spline also produced very low HR estimates; we did not interpret these extreme-tail estimates as a protective biological effect. Overall, the curve does not support a steadily increasing gradient across the entire APAR range.

**Figure 2:**
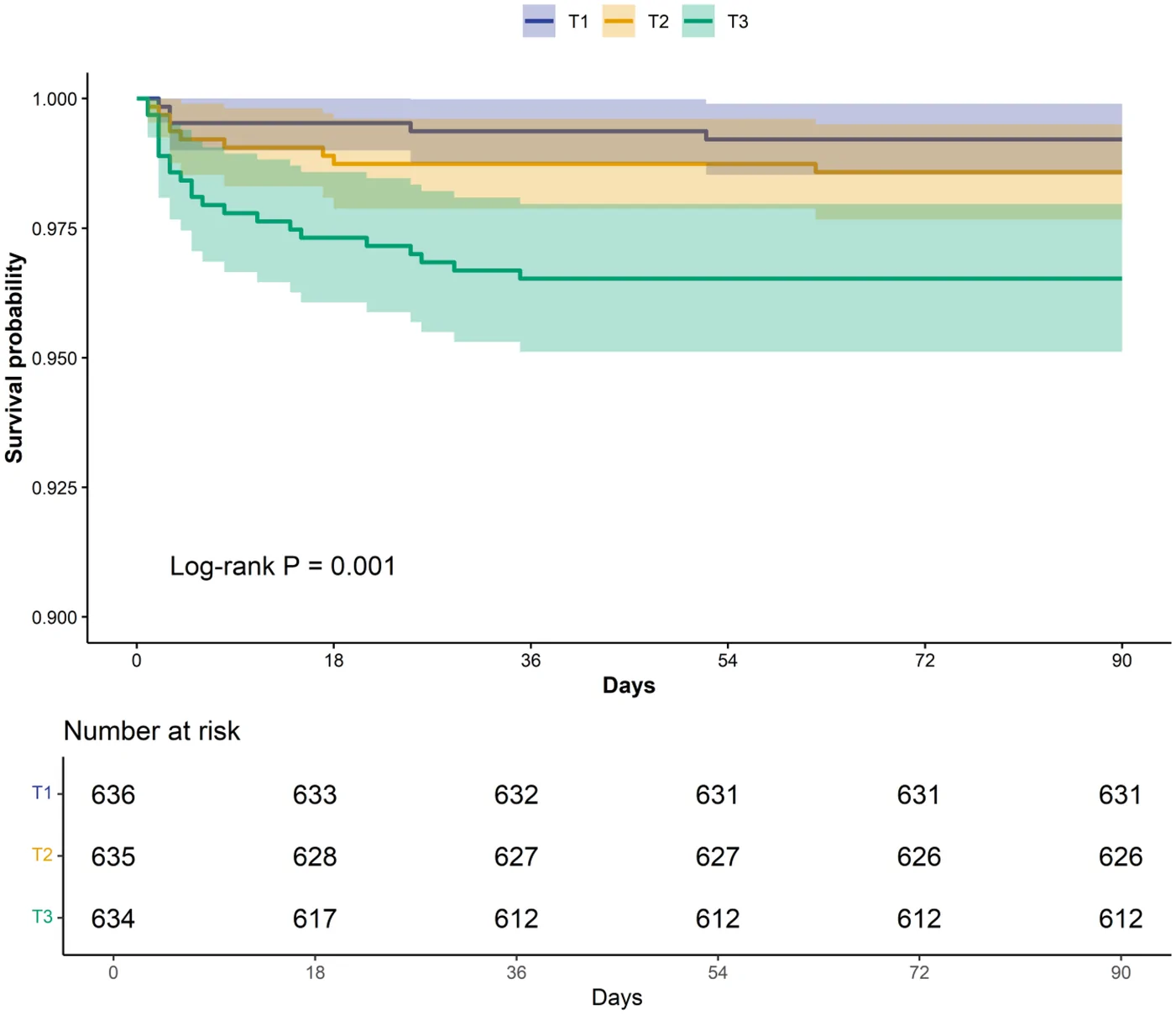
Kaplan–Meier survival curves for 90-day all-cause mortality according to APAR tertiles. The table below the curves shows the number at risk. Log-rank *P* = 0.001. Tertiles are shown in the main survival figure to avoid relying on the outcome-derived Youden cutoff. The time-to-event cohort included 636, 635, and 634 patients in T1, T2, and T3, respectively; one T3 patient with recorded 90-day death lacked an exact event time and was excluded from Kaplan–Meier analysis.

**Figure 3:**
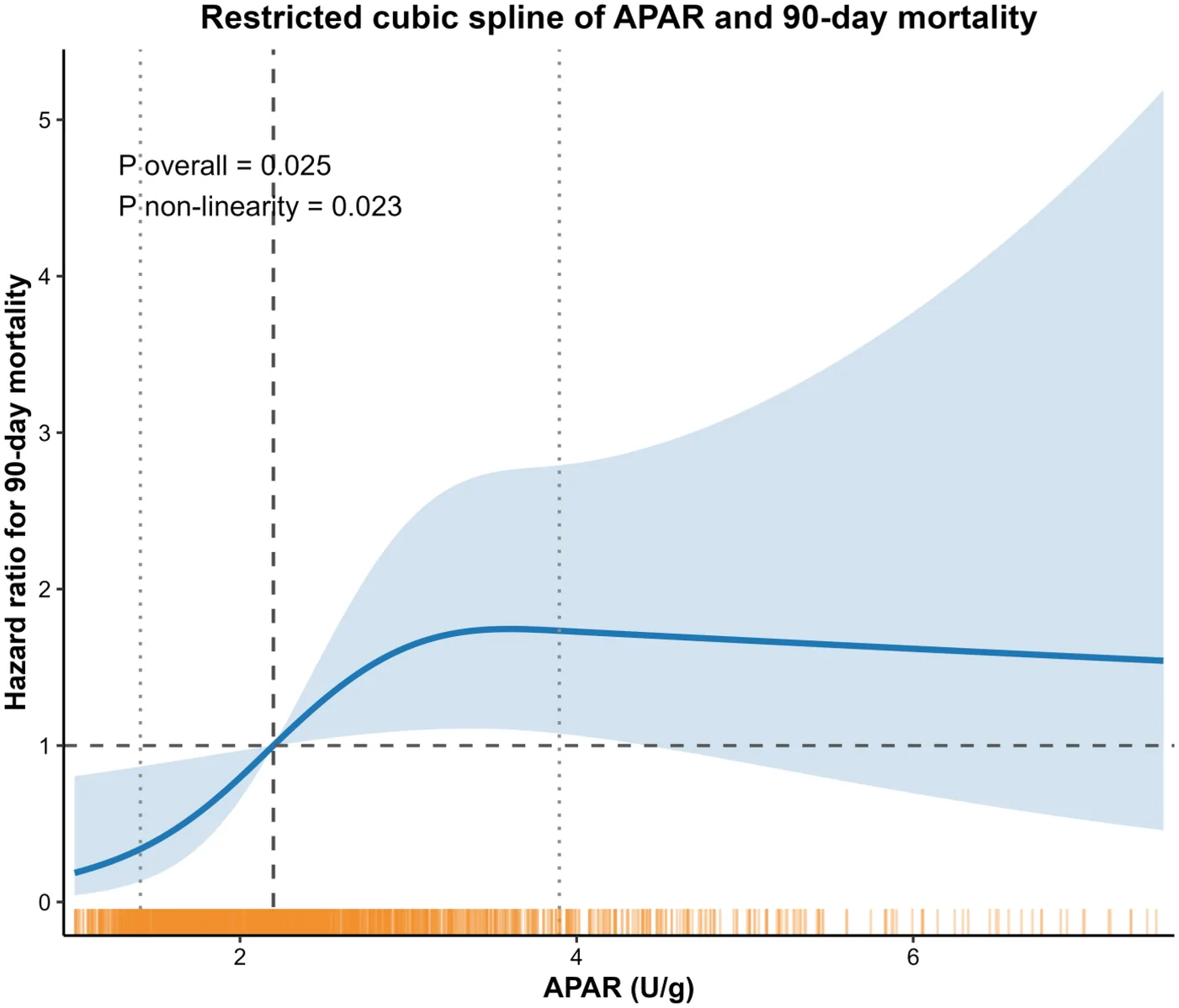
Adjusted restricted cubic spline of APAR and 90-day all-cause mortality. Knots were placed at the 10th, 50th, and 90th percentiles (1.408, 2.198, and 3.897 U/g), with 2.198 U/g as the reference. The model used the primary clinical adjustment set with the eight-category ordinal age score and NYHA stratification. Likelihood-ratio tests showed an overall APAR association (*χ*^2^ = 7.378, df = 2; *P* = 0.025) and nonlinearity (*χ*^2^ = 5.153, df = 1; *P* = 0.023). The shaded area represents the 95% confidence interval, and the rug shows the distribution of APAR.

### Exploratory cutoff analysis

The same-cohort Youden analysis gave an apparent cutoff of 2.82 U/g. We report this only for descriptive comparison. Ninety-day mortality was 1.01% (14/1,389) below the cutoff and 4.45% (23/517) above it, an absolute risk difference of 3.44 percentage points (95% CI: 1.59–5.29). In the exact-time cohort, incidence rates were 0.113 and 0.492 deaths per 1,000 person-days, respectively. The adjusted cutoff-based HR was 2.823 (95% CI 1.391–5.730; *P* = 0.004). Bootstrap resampling gave a 2.5th-97.5th percentile cutoff range of 2.04–3.01 U/g, and the optimism-corrected Youden J was 0.310 vs. an apparent J of 0.357 (Supplementary Table S4 and Figure S2). These results do not support treating 2.82 U/g as a validated clinical threshold.

### Discrimination and incremental information

APAR had an AUC of 0.698 (95% CI, 0.620–0.776), with sensitivity 62.2% and specificity 73.6% at the exploratory same-cohort cutoff (Figure 4; Table 3). ALP and albumin had AUCs of 0.662 and 0.638. The differences were not statistically significant by DeLong testing (APAR vs. ALP, *P* = 0.128; APAR vs. albumin, *P* = 0.117). The 90-day cumulative/dynamic AUC in the exact-time cohort was 0.696 for APAR, consistent with the fixed-horizon estimate.

**Figure 4:**
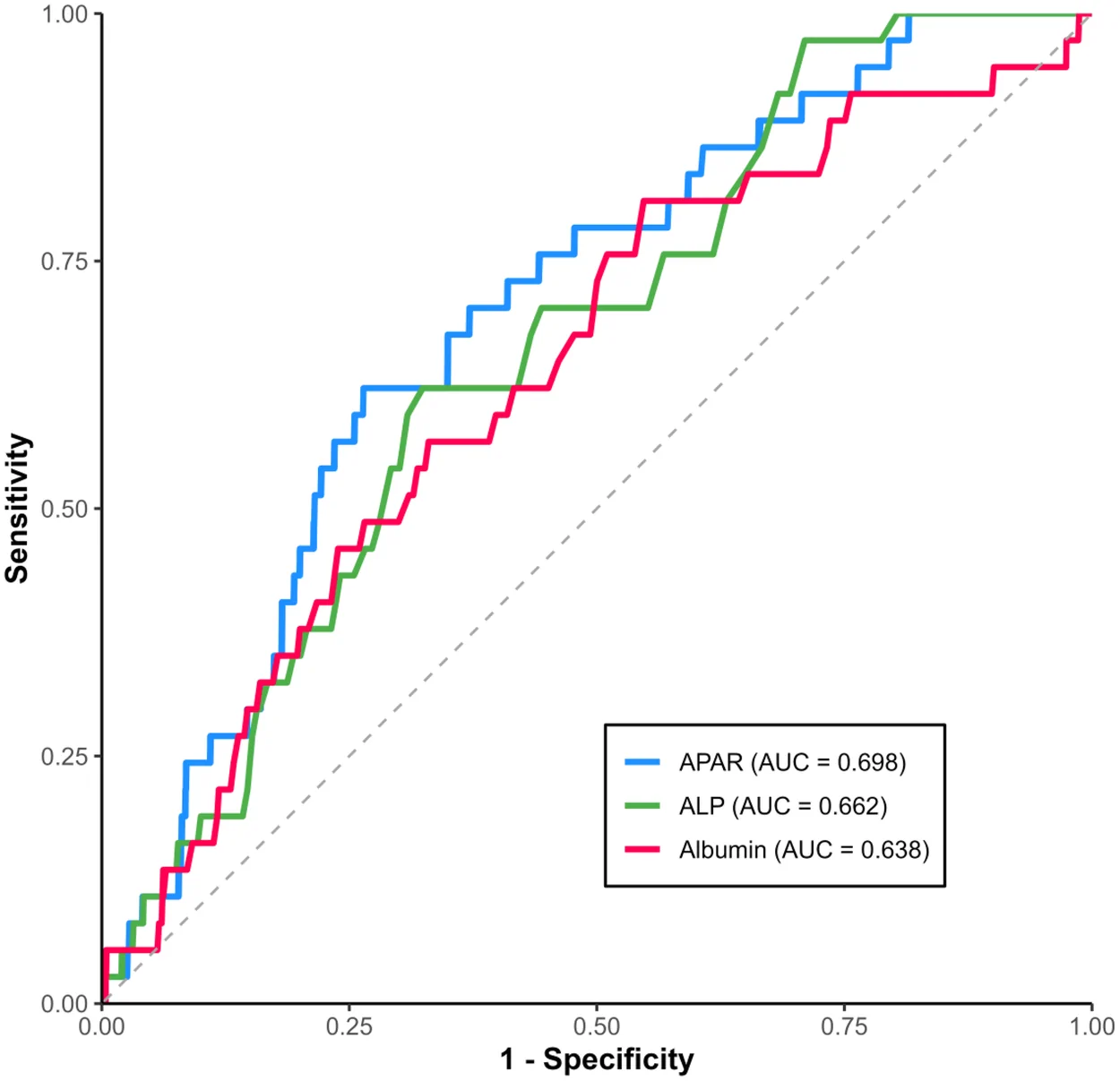
Receiver operating characteristic curves for APAR, ALP, and albumin for 90-day all-cause mortality. Pairwise DeLong tests did not show a statistically significant difference between APAR and ALP (*P* = 0.128) or between APAR and albumin (*P* = 0.117).

**Table 3:** Discrimination, component comparison, and incremental prognostic information of APAR.

Marker	AUC (95% CI)	Cutoff	Sensitivity	Specificity	Comparison
Panel A. Standalone discrimination					
APAR	0.698 (0.620–0.776)	2.82 U/g	0.622	0.736	
ALP	0.662 (0.584–0.740)	93.5 U/L	0.622	0.676	APAR vs. ALP: *Δ*AUC = 0.036; *P* = 0.128
Albumin	0.638 (0.548–0.728)	37.35 g/L	0.811	0.453	APAR vs. albumin: *Δ*AUC = 0.060; *P* = 0.117

Exposure	Adjusted HR (95% CI)	*P* value	*N*/deaths
Panel B. Component Cox models and ratio-constraint test			
ln (APAR)	2.154 (1.059–4.382)	0.034	1,824/36
ln (ALP)	2.098 (0.954–4.612)	0.065	1,824/36
ln (albumin)	0.305 (0.047–1.980)	0.213	1,824/36
Ratio-constraint LRT	*χ*^2^ = 0.108; df = 1	0.742	Separate components vs. constrained ratio

Metric	Clinical model	Clinical model + APAR	Increment/statistic	Internal validation/95% CI	*P* value
Panel C. Incremental prognostic information					
Harrell C-index	0.746	0.764	*Δ* = 0.018	Bootstrap 95% CI −0.0037 to 0.0398	0.104
90-day AUC	0.817	0.834	*Δ* = 0.018	Bootstrap 95% CI 0.0005 to 0.0366	0.038
Likelihood-ratio test			χ^2^ = 4.230		0.040
IDI			0.0019	Bootstrap 95% CI −0.0035 to 0.0252	
Calibration: mean predicted risk	0.0197	0.0196	Observed risk = 0.0197	Grouped calibration curves broadly overlapped	
Brier score	0.018465	0.018504	*Δ* = 0.000039	Smaller is better; change was negligible	
Decision-curve analysis	Reference	Compared with clinical model	Max gain = 0.002730	Median net-benefit gain = 0.000720	
Continuous NRI (secondary)			0.179	Bootstrap 95% CI −0.129 to 0.577	

Component-model HRs use the same primary clinical adjustment set with the eight-category ordinal age score and NYHA stratification. The likelihood-ratio test compares an unrestricted ln(ALP) + ln(albumin) model with the constrained ratio model. Incremental analyses compare the clinical model with the same model plus standardized ln(APAR). IDI, calibration, Brier score, and decision-curve findings are interpreted together; continuous NRI is presented last as a secondary metric.

In separate adjusted component models, ln(APAR) was associated with mortality (HR, 2.154 per 1-unit increase, 95% CI, 1.059–4.382; *P* = 0.034), whereas the intervals for ln(ALP) (HR, 2.098, 95% CI, 0.954–4.612; *P* = 0.065) and ln(albumin) (HR, 0.305, 95% CI, 0.047–1.980; *P* = 0.213) crossed the null. The likelihood-ratio test comparing separate ln(ALP) and ln(albumin) coefficients with the constrained ratio model was not significant (*χ*^2^ = 0.108, df = 1; *P* = 0.742). Thus, the data did not establish that APAR is statistically superior to its components (Table 3; Supplementary Table S5).

Adding APAR to the clinical model increased the C-index from 0.746 to 0.764 (*Δ* = 0.018; bootstrap 95% CI, −0.0037 to 0.0398; *P* = 0.104) and the 90-day AUC from 0.817 to 0.834 (*Δ* = 0.018; bootstrap 95% CI, 0.0005–0.0366; DeLong *P* = 0.038). The likelihood-ratio test was *P* = 0.040. In contrast, IDI was only 0.0019 (95% CI, −0.0035 to 0.0252), mean predicted risk was essentially unchanged (0.0197 vs. 0.0196; observed risk 0.0197), Brier scores were nearly identical, and decision-curve gains were small (maximum net-benefit gain 0.00273; median 0.00072). Continuous NRI was 0.179 but imprecise (95% CI: −0.129 to 0.577) and was therefore treated as secondary. Taken together, these results indicate statistically detectable change in some metrics but little consistent evidence of clinically meaningful incremental prediction (Table 3; Supplementary Figures S3–S4).

### Sensitivity and subgroup analyses

The primary estimate was similar after additional adjustment for HF treatment burden (HR, 1.345, 95% CI, 1.003–1.803; *P* = 0.047) or diuretic use (HR, 1.382, 95% CI, 1.027–1.860; *P* = 0.033). Additional liver-related analyses yielded estimates above 1 but with varying precision; for example, adjustment for GGT and total bilirubin gave HR, 1.534 (95% CI, 1.092–2.154; *P* = 0.013), whereas adjustment for AST and ALT gave HR, 1.254 (95% CI, 0.903–1.742; *P* = 0.177). In the LVEF-measured subset, only 612 patients and 9 deaths were available, producing an imprecise estimate (HR, 1.368, 95% CI, 0.754–2.481; *P* = 0.302). Multiple imputation gave HR, 1.391 (95% CI, 1.035–1.870; *P* = 0.029). On the same hemoglobin-complete subset (*n* = 1,809; 36 deaths), adding hemoglobin changed the APAR estimate little (HR, 1.369, 95% CI, 1.017–1.842; *P* = 0.038), while hemoglobin itself was not associated with mortality after adjustment (HR, 0.963 per 10 g/L, *P* = 0.621). Ridge shrinkage attenuated the APAR coefficient (penalized HR 1.313 at *λ*min and approximately 1.00 at *λ*1se) (Supplementary Table S6).

A 28-day sensitivity model included 1,825 patients and 32 deaths; the adjusted HR per 1-SD increase in ln(APAR) was 1.357 (95% CI, 0.992–1.855; *P* = 0.056), which was directionally consistent but did not meet the conventional 0.05 threshold (Supplementary Table S6 and Figure S6). In the strict post-discharge complete-case cohort, 1,797 patients contributed 15 definite later-day deaths. The crude HR was 1.735 (95% CI, 1.239–2.431; *P* = 0.001), while the fully adjusted HR was 1.460 (95% CI, 0.931–2.289; *P* = 0.099). Because the fully adjusted post-discharge model had only 2.14 events per estimated coefficient, we regard it as exploratory and imprecise (Supplementary Table S8 and Figure S7). In exploratory subgroup analyses, no interaction *P* value was below 0.05 after multiplicity-aware interpretation; all FDR-adjusted q values were at least 0.792 (Supplementary Table S7 and Figure S5).

## Discussion

In this cohort of hospitalized patients with HF, higher admission ln(APAR) was associated with a higher risk of recorded early all-cause mortality within 90 days of admission after parsimonious adjustment. The effect remained modest: the adjusted HR was 1.375 per 1-SD increase in ln(APAR), and only 36 deaths contributed exact event times to the Cox model. The raw APAR scale yielded HR 1.146 per 1 U/g with *P* = 0.072, demonstrating that statistical significance depended on parameterization. The association was also nonlinear, with most of the rise in estimated risk occurring in the lower-to-middle APAR range and little further increase at higher values. These features argue against describing APAR as a simple, scale-invariant dose-dependent severity marker.

Our findings are broadly consistent with reports linking higher APAR to adverse outcomes in coronary artery disease, sepsis, and chronic kidney disease (27–29). The HF setting, however, is different. ALP and albumin can both move with venous congestion, liver dysfunction, hemodilution, inflammation, nutritional compromise, renal disease, and malignancy. In our data, higher APAR coincided with higher BNP and GGT, lower sodium and eGFR, and more NYHA IV disease. The APAR association attenuated after adjustment for these severity-related factors. For that reason, we interpret APAR as a possible summary of systemic and congestive illness rather than as evidence of a distinct nutrition-inflammation pathway.

The comparison with ALP and albumin also constrained the interpretation. APAR had the numerically highest standalone AUC, but DeLong testing did not show a significant difference from either component. In adjusted component models, ln(APAR) reached conventional statistical significance whereas the component intervals crossed the null; however, the ratio-constraint likelihood-ratio test was also negative (*P* = 0.742). These results neither prove that the ratio is better nor make it redundant. They suggest that APAR is a compact way to combine two routinely available measurements, but any claimed advantage over ALP or albumin requires confirmation in a larger independent cohort.

The same caution applies to prediction. Adding APAR changed the C-index and 90-day AUC by only 0.018. Although the AUC difference and likelihood-ratio test reached conventional statistical significance, the C-index increment was not significant, IDI was very small and imprecise, calibration and Brier score were essentially unchanged, and decision-curve gains were minimal. Continuous NRI was also imprecise and was interpreted as a secondary metric. The overall pattern therefore supports, at most, limited incremental prognostic information rather than clinically meaningful improvement in a prediction model.

The nonlinear shape and the cutoff analysis point in the same direction. The spline rose across the lower-to-middle APAR range and then plateaued, while the Youden cutoff varied from about 2.04 to 3.01 U/g across bootstrap samples. This instability is expected when a threshold is optimized in the same cohort in which its performance is evaluated. The value of 2.82 U/g is therefore useful only as a descriptive feature of this dataset and should not be used as a universal clinical boundary.

We also considered whether the association could be explained by non-HF causes of abnormal ALP or albumin. Adjustment for treatment burden, liver disease, malignancy, liver biochemistry, and hemoglobin did not materially reverse the direction of the APAR estimate, although several analyses became imprecise as events were lost or additional covariates were introduced. In the hemoglobin-adjusted analysis, the APAR estimate remained similar (HR, 1.369) on the same hemoglobin-complete subset. LVEF could be examined in only 612 patients with 9 deaths, so that sensitivity analysis cannot determine whether the association is independent of HF phenotype or systolic function. Residual confounding by congestion, liver or bone disease, malignancy, medication intensity, frailty, and other unmeasured factors remains plausible.

Mortality-timing analysis showed that the recorded 90-day endpoint was not dominated by in-hospital deaths. Only 9 of 37 recorded 90-day deaths were confirmed during the index hospitalization. Among patients discharged without a recorded in-hospital death, 15 deaths occurred on a later calendar date after discharge, 12 were recorded on the same calendar day as discharge and could not be ordered within that day, and one lacked exact timing. The source descriptor reported 14 in-hospital deaths among 2,008 patients, compared with 9 among 1,906 patients in the APAR cohort. The strict post-discharge analysis was directionally consistent with the main result but imprecise after full adjustment, with only 15 definite later-day events. The 28-day estimate was likewise directionally similar but imprecise. These findings support interpreting the endpoint as recorded early mortality within a 90-day window rather than as evidence of a stable long-term prognostic effect.

Several limitations remain. The retrospective single-center design precludes causal inference and limits generalizability. Only 37 90-day deaths were recorded, and one lacked an exact event time; the strict post-discharge model contained only 15 definite later-day events and was especially vulnerable to overfitting. The public release does not contain contact-level follow-up logs or an explicit unresolved-status field, so survival status could not be independently verified for every nonfatal record. The E-value was 2.09 for the point estimate but only 1.18 for the confidence-limit value closest to the null, indicating that the lower bound of the association is not highly robust to modest unmeasured confounding; moreover, E-values do not directly address outcome misclassification. Patients without ALP or albumin differed from included patients on several baseline characteristics, so selection bias is possible. The released anthropometric fields also contain implausible extreme weight/height-derived BMI values; we therefore treated BMI as a quality-control variable rather than an inferential covariate. LVEF and AST were substantially missing, and the public documentation does not provide assay-specific details or ALP isoenzymes. Finally, APAR was measured once at baseline, so we could not examine whether changes with decongestion or recovery carry additional information.

## Conclusion

Higher admission ln(APAR) was associated with greater recorded early all-cause mortality within 90 days of admission in this hospitalized HF cohort, but the association was modest, nonlinear, scale-dependent, and based on few events. The strict post-discharge analysis was directionally consistent but imprecise. APAR was not statistically superior to ALP or albumin and added only limited incremental prognostic information to the clinical model. The same-cohort 2.82 U/g cutoff should remain exploratory until the association and any useful threshold are validated externally.

## Data Availability

The data analyzed in this study are available from the restricted-access PhysioNet resource ‘Hospitalized patients with heart failure: integrating electronic healthcare records and external outcome data,’ version 1.3. Eligible researchers may request access through PhysioNet subject to its data-use requirements: https://physionet.org/content/heart-failure-zigong/1.3/.
