# Supplementary material for: Association between the alkaline phosphatase-to-albumin ratio and recorded early all-cause mortality within 90 days of admission in patients with heart failure: a retrospective cohort study in a Chinese population
Source: Front Cardiovasc Med. 2026 Sep 16;13:1936612. doi: 10.3389/fcvm.2026.1936612 (PMC13623932; doi:10.3389/fcvm.2026.1936612)
Supplement: Supplementary file 1 [file Supplementaryfile1.docx]

**Supplementary Material**

**Supplementary Table S1. Missingness in the APAR cohort (N=1,906)**

| **Variable** | **Missing, n** | **Missing, %** |
| --- | --- | --- |
| **Exposure and outcome** | | |
| ALP | 0 | 0 |
| Albumin | 0 | 0 |
| APAR | 0 | 0 |
| 90-day mortality status | 0 | 0 |
| **Demographic and clinical variables** | | |
| Age category | 0 | 0 |
| Sex | 0 | 0 |
| Weight | 0 | 0 |
| Heart rate | 0 | 0 |
| SBP | 0 | 0 |
| DBP | 0 | 0 |
| NYHA class | 0 | 0 |
| CCI | 5 | 0.26 |
| GCS | 0 | 0 |
| Diabetes | 0 | 0 |
| Chronic pulmonary disease | 0 | 0 |
| Chronic kidney disease | 2 | 0.1 |
| **Laboratory and imaging variables** | | |
| WBC | 21 | 1.1 |
| Hemoglobin | 22 | 1.15 |
| Platelets | 21 | 1.1 |
| Neutrophils | 21 | 1.1 |
| BUN | 13 | 0.68 |
| Creatinine | 13 | 0.68 |
| eGFR | 51 | 2.68 |
| Potassium | 7 | 0.37 |
| Sodium | 7 | 0.37 |
| Chloride | 7 | 0.37 |
| BNP | 25 | 1.31 |
| AST | 226 | 11.86 |
| ALT | 0 | 0 |
| GGT | 0 | 0 |
| Total bilirubin | 0 | 0 |
| LVEF | 1294 | 67.89 |
| **Additional comorbidities and medications** | | |
| Liver disease | 1 | 0.05 |
| Any malignancy | 0 | 0 |
| Beta-blocker | 1 | 0.05 |
| Diuretic | 1 | 0.05 |
| Nitrate | 1 | 0.05 |
| Vasoactive drug | 1 | 0.05 |
| Digoxin | 1 | 0.05 |
|  |  |  |
| **ALP, albumin, and APAR have no missing values in this table because calculable APAR was required for inclusion in the APAR cohort.** | | |

**Supplementary Table S2. Comparison of patients with and without calculable APAR.**

| **Variable** | **Included (n=1,906)** | **Excluded (n=102)** | **P value** | **SMD** |
| --- | --- | --- | --- | --- |
| **Demographic and clinical variables** | | | | |
| Weight | 50.00 (45.00, 60.00) | 49.00 (45.00, 58.00) | 0.423 | 0.019 |
| Heart rate | 82.50 (70.00, 98.00) | 80.00 (70.00, 95.75) | 0.662 | 0.034 |
| SBP | 130.00 (114.00, 148.00) | 122.00 (110.00, 140.00) | 0.015 | 0.263 |
| DBP | 76.00 (66.00, 85.00) | 74.00 (62.00, 84.75) | 0.346 | 0.13 |
| Male sex | 797 (41.8%) | 48 (47.1%) | 0.305 | -0.106 |
| GCS | 15.00 (15.00, 15.00) | 15.00 (15.00, 15.00) | 0.193 | 0.136 |
| **Laboratory variables** | | | | |
| WBC | 6.50 (5.09, 8.68) | 6.56 (4.98, 8.29) | 0.771 | 0.031 |
| Hemoglobin | 117.00 (101.00, 131.00) | 119.50 (105.75, 137.25) | 0.117 | -0.18 |
| Platelets | 135 (100, 176) | 139 (107, 189) | 0.162 | -0.164 |
| Neutrophils | 4.83 (3.61, 6.83) | 4.72 (3.47, 6.43) | 0.53 | 0.059 |
| BUN | 8.05 (5.91, 11.56) | 7.53 (5.75, 10.76) | 0.283 | 0.126 |
| Creatinine | 87.40 (65.00, 123.70) | 79.50 (60.25, 110.22) | 0.045 | 0.172 |
| eGFR | 64.43 (41.12, 89.75) | 70.19 (47.60, 98.73) | 0.036 | -0.265 |
| Sodium | 139.00 (136.00, 141.40) | 137.90 (134.12, 140.30) | 0.005 | 0.27 |
| BNP | 766.57 (315.86, 1760.45) | 453.12 (183.59, 1286.58) | 0.006 | 0.17 |
| **Comorbidities and outcome** | | | | |
| CCI | 2.00 (1.00, 2.00) | 2.00 (1.00, 2.00) | 0.7 | 0.009 |
| Diabetes | 441 (23.1%) | 25 (24.5%) | 0.719 | -0.032 |
| Chronic pulmonary disease | 224 (11.8%) | 9 (8.8%) | 0.43 | 0.097 |
| Chronic kidney disease | 449 (23.6%) | 25 (24.5%) | 0.811 | -0.022 |
| Liver disease | 81 (4.3%) | 3 (2.9%) | 0.798 | 0.07 |
| Any malignancy | 36 (1.9%) | 4 (3.9%) | 0.142 | -0.121 |
| 90-day mortality | 37 (1.9%) | 5 (4.9%) | 0.059 | -0.163 |
|  |  |  |  |  |
| **Excluded patients lacked ALP and/or albumin and therefore could not have APAR calculated. P values and SMDs are descriptive.** | | | | |

**Supplementary Table S3. Proportional-hazards assumption for the primary adjusted Cox model.**

| **Variable** | **χ²** | **df** | **P value** |
| --- | --- | --- | --- |
| ln(APAR), per 1 SD | 0.881 | 1 | 0.348 |
| Age category, ordinal score 1-8 | 0.991 | 1 | 0.320 |
| Male sex | 1.181 | 1 | 0.277 |
| log(BNP), standardized | 1.034 | 1 | 0.309 |
| eGFR, standardized | 0.002 | 1 | 0.963 |
| Sodium, standardized | 3.744 | 1 | 0.053 |
| CCI | 0.704 | 1 | 0.401 |
| GLOBAL | 8.092 | 7 | 0.325 |

Tests were based on scaled Schoenfeld residuals. The primary model used the eight released age categories as an ordinal score from 1 to 8 and included sex, log(BNP), eGFR, sodium, CCI, and standardized ln(APAR), with stratification by NYHA class.

**Supplementary Table S4. Exploratory Youden-cutoff analysis (APAR threshold 2.82 U/g)**

| **Panel A. Group-specific 90-day outcomes** | | | | | | |
| --- | --- | --- | --- | --- | --- | --- |
| Group | N (fixed status) | 90-day deaths | Mortality | Person-days | Exact-time deaths | Incidence / 1,000 person-days |
| Low APAR | 1389 | 14 | 1.01% | 123,953 | 14 | 0.113 |
| High APAR | 517 | 23 | 4.45% | 44,682 | 22 | 0.492 |
| **Panel B. Cutoff stability and exploratory association** | | | | | | |
| Metric | Result |  |  |  |  |  |
| Apparent Youden cutoff | 2.82 U/g |  |  |  |  |  |
| Bootstrap median cutoff | 2.82 U/g |  |  |  |  |  |
| Bootstrap 2.5th-97.5th percentile range | 2.04-3.01 U/g |  |  |  |  |  |
| Sensitivity at apparent cutoff | 62.20% |  |  |  |  |  |
| Specificity at apparent cutoff | 73.60% |  |  |  |  |  |
| Apparent Youden J | 0.357 |  |  |  |  |  |
| Mean bootstrap optimism | 0.047 |  |  |  |  |  |
| Optimism-corrected Youden J | 0.310 |  |  |  |  |  |
| Absolute 90-day risk difference (high - low) | 3.44 percentage points (95% CI 1.59-5.29) |  |  |  |  |  |
| Adjusted high-versus-low Cox HR | 2.823 (95% CI 1.391-5.730); P=0.004 |  |  |  |  |  |

The threshold was derived from the same 90-day outcome data and is therefore exploratory. Bootstrap results quantify sampling variability and optimism; 2.82 U/g should not be interpreted as a validated universal clinical cutoff.

**Supplementary Table S5. Component Cox models and ratio-constraint test**

| **Panel A. Separate component Cox models** | | | | |
| --- | --- | --- | --- | --- |
| Exposure | N | Deaths | Adjusted HR (95% CI) | P value |
| ln(APAR) | 1824 | 36 | 2.154 (1.059-4.382) | 0.034 |
| ln(ALP) | 1824 | 36 | 2.098 (0.954-4.612) | 0.065 |
| ln(albumin) | 1824 | 36 | 0.305 (0.047-1.980) | 0.213 |
| **Panel B. Ratio-constraint likelihood-ratio test** | | | | |
| Comparison | LR χ² | df | P value |  |
| Unrestricted ln(ALP)+ln(albumin) vs constrained ln(APAR) | 0.108 | 1 | 0.742 |  |

Component-model HRs are expressed per 1-unit increase on the natural-log scale and use the primary clinical adjustment set with the eight-category ordinal age score and NYHA stratification.

**Supplementary Table S6. Sensitivity analyses for the association of ln(APAR) per 1 SD with mortality**

| **Analysis** | **N** | **Events** | **Measure** | **Effect estimate (95% CI)** | **P value / note** |
| --- | --- | --- | --- | --- | --- |
| Primary complete-case; NYHA-stratified | 1824 | 36 | HR | 1.375 (1.024-1.846) | 0.034 |
| + HF treatment count | 1823 | 36 | HR | 1.345 (1.003-1.803) | 0.047 |
| + Diuretic | 1823 | 36 | HR | 1.382 (1.027-1.860) | 0.033 |
| + Liver disease + malignancy | 1824 | 36 | HR | 1.331 (0.995-1.782) | 0.054 |
| + GGT + total bilirubin | 1824 | 36 | HR | 1.534 (1.092-2.154) | 0.013 |
| + AST + ALT | 1625 | 32 | HR | 1.254 (0.903-1.742) | 0.177 |
| + AST + ALT + GGT + total bilirubin, exploratory | 1625 | 32 | HR | 1.453 (0.998-2.114) | 0.051 |
| Exclude liver disease | 1745 | 29 | HR | 1.361 (0.974-1.900) | 0.071 |
| Exclude malignancy | 1789 | 34 | HR | 1.304 (0.960-1.771) | 0.090 |
| Exclude liver disease and malignancy | 1714 | 28 | HR | 1.329 (0.944-1.871) | 0.104 |
| + LVEF measured subset, reduced exploratory model | 612 | 9 | HR | 1.368 (0.754-2.481) | 0.302 |
| Multiple imputation (20 datasets) | 1905 | 36 | HR | 1.391 (1.035-1.870) | 0.029 |
| Hemoglobin-complete subset + hemoglobin | 1809 | 36 | HR | 1.369 (1.017-1.842) | 0.038 |
| Hemoglobin, per 10 g/L in same model | 1809 | 36 | HR | 0.963 (0.831-1.117) | 0.621 |
| 28-day sensitivity | 1825 | 32 | HR | 1.357 (0.992-1.855) | 0.056 |
| Ridge Cox, λmin | 1824 | 36 | Penalized HR | 1.313 | λ=0.00857 |
| Ridge Cox, λ1se | 1824 | 36 | Penalized HR | 1.000 | λ=13.329 |

Unless otherwise stated, effect estimates are HRs per 1-SD increase in ln(APAR) and use the primary clinical covariates with NYHA stratification. The hemoglobin comparison was performed on the same hemoglobin-complete subset. The LVEF subset and 28-day analysis were event-limited; ridge estimates illustrate shrinkage rather than conventional hypothesis tests.

**Supplementary Table S7. Exploratory subgroup analyses**

| **Subgroup** | **Level** | **N / events** | **HR per 1-SD ln(APAR)** | **P for interaction** | **FDR q** |
| --- | --- | --- | --- | --- | --- |
| Overall | All patients | 1905 / 36 | 1.64 (1.29-2.07) |  |  |
| Sex | Female | 1109 / 18 | 1.49 (0.98-2.27) | 0.638 | 0.902 |
|  | Male | 796 / 18 | 1.68 (1.28-2.21) |  |  |
| CCI | 0-2 | 1442 / 20 | 1.67 (1.24-2.24) | 0.739 | 0.902 |
|  | ≥3 | 458 / 16 | 1.53 (1.00-2.32) |  |  |
| Diabetes | No | 1464 / 26 | 1.69 (1.31-2.17) | 0.624 | 0.902 |
|  | Yes | 441 / 10 | 1.44 (0.80-2.59) |  |  |
| NYHA class | II-III | 1323 / 12 | 1.92 (1.41-2.61) | 0.132 | 0.792 |
|  | IV | 582 / 24 | 1.33 (0.94-1.87) |  |  |
| Chronic pulmonary disease | No | 1681 / 33 | 1.64 (1.29-2.09) | 0.854 | 0.902 |
|  | Yes | 224 / 3 | 1.50 (0.56-3.97) |  |  |
| Chronic kidney disease | No | 1454 / 16 | 1.62 (1.14-2.29) | 0.902 | 0.902 |
|  | Yes | 449 / 20 | 1.57 (1.10-2.24) |  |  |

Subgroup-specific HRs were estimated from unadjusted Cox models and are presented per 1-SD increase in ln(APAR). Interaction P values were obtained from the corresponding unadjusted interaction models and adjusted using the Benjamini-Hochberg false-discovery-rate procedure. These analyses were exploratory. Age was not included as a binary subgroup because the released age variable comprised eight ordered categories and was modeled using the full ordinal structure in the primary analysis.

**Supplementary Table S8. In-hospital mortality, discharge timing, length of stay, and strict post-discharge sensitivity analysis**

| **Panel A. Mortality disposition within the 90-day window** | | | | |
| --- | --- | --- | --- | --- |
| Category | N | Denominator / percentage | Interpretation |  |
| Recorded 90-day deaths | 37 | 37 / 1.94% of cohort | Primary fixed-horizon outcome |  |
| Confirmed in-hospital deaths | 9 | 9/1906 (0.47%) | hospital_outcome == Dead |  |
| Discharged without recorded in-hospital death | 1897 | 1897/1906 (99.53%) | Includes discharge against medical advice |  |
| 90-day deaths among these discharged patients | 28 | 28/1897 (1.48%) | Includes later-day, same-day, and missing-time deaths |  |
| Definite later-day post-discharge deaths | 15 | 15/1897 (0.79%) | death date strictly later than discharge date |  |
| Same-day deaths | 12 | 12/1897 (0.63%) | Within-day order unresolved |  |
| Exact death time missing after non-in-hospital discharge | 1 | 1/1897 (0.05%) | Timing unclassified |  |
| Death-before-discharge temporal conflicts | 0 | 0 | No such conflicts |  |
| **Panel B. Index-admission length of stay** | | | | |
| Group | N | Median (IQR), days | Mean ± SD, days |  |
| Overall APAR cohort | 1906 | 8.0 (6.0-10.0) | 9.4 ± 8.1 |  |
| Discharged without recorded in-hospital death | 1897 | 8.0 (6.0-10.0) | 9.4 ± 8.0 |  |
| Confirmed in-hospital deaths | 9 | 17.0 (5.0-26.0) | 18.9 ± 18.9 |  |
| **Panel C. Strict post-discharge Cox analysis** | | | | |
| Model | N / events | HR per 1-SD ln(APAR) | 95% CI | P value |
| Crude | 1797 / 15 | 1.735 | 1.239-2.431 | 0.001 |
| Primary-covariate adjusted | 1797 / 15 | 1.460 | 0.931-2.289 | 0.099 |
| **Panel D. Sparse-event diagnostic** | | | | |
| Estimated coefficients in adjusted post-discharge model | 7 |  |  |  |
| Events per estimated coefficient | 2.14 |  |  |  |
| Interpretation | Sparse-event sensitivity analysis; adjusted estimate should be interpreted cautiously |  |  |  |

Confirmed in-hospital death was defined strictly from the released hospital_outcome field. The strict post-discharge analysis excluded same-day deaths, deaths without exact timing, and discharge-status quality-control conflicts. Because the release records timing by calendar day, same-day deaths could not be assigned as occurring before or after discharge within that day. The source database descriptor reported 14 in-hospital deaths among 2,008 patients (0.70%), compared with 9 among 1,906 patients (0.47%) in the APAR cohort.

**Supplementary Table S9. Released age categories, event distribution, and age parameterization**

| **Panel A. Released age categories and 90-day deaths in the complete-case Cox cohort** | | | | |
| --- | --- | --- | --- | --- |
| Age category | Ordinal score | APAR cohort N (%) | Cox cohort N | 90-day deaths |
| (21,29] | 1 | 4 (0.2%) | 4 | 0 |
| (29,39] | 2 | 12 (0.6%) | 12 | 0 |
| (39,49] | 3 | 50 (2.6%) | 49 | 1 |
| (49,59] | 4 | 102 (5.4%) | 96 | 1 |
| (59,69] | 5 | 347 (18.2%) | 331 | 6 |
| (69,79] | 6 | 681 (35.7%) | 652 | 14 |
| (79,89] | 7 | 617 (32.4%) | 591 | 13 |
| (89,110] | 8 | 93 (4.9%) | 89 | 1 |

Age was modeled as a single ordinal score from 1 to 8. A conventional seven-degree-of-freedom categorical specification produced unstable coefficient estimates because of sparse and zero-event age categories.

**Supplementary Table S10. E-value sensitivity analysis for the primary ln(APAR) association**

| **Exposure** | **Adjusted HR** | **95% CI** | **E-value for point estimate** | **CI limit closest to null** | **E-value for CI limit** |
| --- | --- | --- | --- | --- | --- |
| ln(APAR), per 1 SD | 1.375 | 1.024-1.846 | 2.092 | 1.024 | 1.180 |

The E-value was calculated using the adjusted hazard ratio as an approximate risk ratio. It quantifies the strength of association that an unmeasured confounder would need with both exposure and outcome to explain away the observed association under the E-value assumptions; it does not directly quantify outcome misclassification or incomplete follow-up ascertainment.

**Supplementary Table S11. Verification of weight and BMI distributions**

| **Variable** | **Group** | **N** | **Median (IQR)** | **Minimum** | **Maximum** |
| --- | --- | --- | --- | --- | --- |
| Weight, kg | Overall | 1906 | 50.00 (45.00-60.00) | 0 | 115 |
|  | T1 | 636 | 51.00 (45.00-60.00) | 30 | 115 |
|  | T2 | 635 | 50.00 (45.00-60.00) | 0 | 96 |
|  | T3 | 635 | 50.00 (45.00-60.00) | 0 | 92 |
| BMI, kg/m² | Overall | 1906 | 20.78 (18.49-23.44) | 0 | 404.08 |
|  | T1 | 636 | 21.22 (18.73-24.01) | 13.32 | 138.89 |
|  | T2 | 635 | 20.72 (18.43-23.44) | 0 | 404.08 |
|  | T3 | 635 | 20.55 (18.37-23.16) | 0 | 404.08 |

BMI was examined as an anthropometric quality-control measure; however, implausible extreme values were present in the released anthropometric data, and BMI was therefore not used for inferential analyses or primary model adjustment.

**Supplementary Figure S1. Distribution of exact recorded times to death within 90 days.**

**Supplementary Figure S2. Bootstrap distribution and optimism assessment of the exploratory Youden cutoff.**

**Supplementary Figure S3. Calibration of the clinical model and clinical model plus APAR at 90 days.**

**Supplementary Figure S4. Decision-curve analysis at 90 days.**

**Supplementary Figure S5. Exploratory subgroup forest plot for 90-day all-cause mortality. Hazard ratios are shown per 1-SD increase in ln(APAR). Interaction tests were exploratory.**

**Supplementary Figure S6. Kaplan-Meier sensitivity analysis for 28-day all-cause mortality according to the exploratory APAR cutoff.**

**Supplementary Figure S7. Strict post-discharge Kaplan-Meier survival curves according to APAR group. Same-day deaths and records without resolvable post-discharge timing were excluded from this sensitivity analysis. Follow-up was measured from discharge but remained bounded by 90 days after the index admission; therefore, the maximum post-discharge follow-up varied according to the length of the index hospitalization.**
